# Exogenous melatonin confers cold tolerance in rapeseed (*Brassica napus* L.) seedlings by improving antioxidants and genes expression

**DOI:** 10.1080/15592324.2022.2129289

**Published:** 2022-10-07

**Authors:** Yan Lei, Huang He, Ali Raza, Zeng Liu, Ding Xiaoyu, Wang Guijuan, Lv Yan, Cheng Yong, Zou Xiling

**Affiliations:** a Key Laboratory Biology and Genetic Improvement of Oil Crops, Oil Crops Research Institute, Chinese Academy of Agricultural Sciences (CAAS), Ministry of Agriculture, Wuhan, China; b Seed Administration Bureau of Hubei Province, Wuhan, China

**Keywords:** Cold stress, neurotransmitter, osmoprotectent, physiological indexes, transcriptional level

## Abstract

Rapeseed (*Brassica napus* L.) is an important oilseed crop globally. However, its growth and production are significantly influenced by cold stress. To reveal the protective role of exogenous melatonin (MEL) in cold tolerance, rapeseed seedlings were pretreated with different concentrations of MEL before cold stress. The results indicated that the survival rate was increased significantly by the MEL pretreatment under cold stress. Seedlings pretreated with 0.01 g L^−1^ MEL were all survived and were used to analyze the physiological characteristics and the expression level of various genes related to cold tolerance. Under cold stress, exogenous MEL significantly increased the contents of proline, soluble sugar, and soluble protein; while the malondialdehyde content was decreased by exogenous MEL under cold stress. On the other hand, the activities of antioxidant defense enzymes such as catalase, peroxidase, and superoxide dismutase were also significantly enhanced. The results also showed that MEL treatment significantly upregulated the expression of *Cu-SOD, COR6.6* (*cold-regulated), COR15*, and *CBFs* (*C-repeat binding factor*) genes under cold stress. It was suggested exogenous MEL improved the content of osmotic regulatory substances to maintain the balance of cellular osmotic potential under cold stress and improved the scavenging capacity of reactive oxygen species by strengthening the activity of antioxidant enzymes and the cold-related genes expression.

## 1. Introduction

Rapeseed (*Brassica napus* L.) is the most important and widely planted oil crop in China, and its annual planting area is about 100 million mu.^1,2^ The Yangtze River Basin is the main rapeseed-producing area in China, which accounts for about 80% of the total planting area and yield in China.^1,2^ In this area, rapeseed is planted as a rotated crop with rice. With the continuous delay of rice harvest time, the sowing date of rapeseed has been gradually delayed in recent years, and its seedling stage has been delayed until after December.^1–3^ After November, the temperature in the Yangtze River Basin will drop below 15°C, and the lowest temperature can reach −6°C. During this period, low temperature disasters frequently occur.^1,3^ Among different abiotic stresses, cold stress seriously affects rapeseed germination and seedling formation, reduces the germination rate, and leads to wilting and death of plants at the seedling stage.^4–8^ The results of freezing treatment on 226 rapeseed varieties showed that the cold tolerance of all the varieties was generally weak.^9^ Therefore, low-temperature stress has become an important challenge for rapeseed production.

Melatonin (MEL: a plant-based neurotransmitter), also known as N-acetyl-5-methoxytryptamine, was first discovered in the bovine pineal gland.^10^ Studies in plants have shown that MEL regulates plant growth, root morphogenesis, floral transformation, and responses to multiple abiotic stresses,^11–17^ including cold stress.^14,18^ For instance, MEL can alleviate the drought stress of wheat, rapeseed and maize by reducing the malondialdehyde (MDA) contents.^19–21^ In Limonium bicolor, wheat, and cotton, MEL improved salt tolerance by enhancing the antioxidant enzyme activities and the content of soluble sugar.^22–24^ In a recent study, Shi, Liang^25^ found that under low-temperature stress, 100 μmol L^−1^ of exogenous MEL pretreatment significantly reduced the MDA content, ROS level, and electrolyte leakage, and increased the chlorophyll content and antioxidant enzyme system activity, induced *MAPK3, MAPK4*, and *MAPK6* gene expression, decreased plasma membrane peroxidation level, maintained cell membrane integrity and enhanced adaptability of rapeseed seedlings under low temperature.^25^ In conclusion, MEL has been widely used to improve the tolerance against drought, salt, and cold stress. However, in response to cold stress, most studies have been carried out to identify the MEL-induced stress tolerance mechanisms at physiological and biochemical levels in different crop plants;^14,18^ still, little is known about MEL-induced cold tolerance in rapeseed. Thus, there was a dire need to evaluate the positive impact of MEL in rapeseed seedlings at molecular level. Another issue, the selection of optimal MEL dose under varying cold stress conditions has been resolved in the present work.

Therefore, the current study was designed to unravel the positive role of MEL to improve cold stress tolerance in rapeseed seedlings. To select the best performed MEL dose, the seedlings were pretreated with 0, 0.005, 0.0075, 0.01, 0.015, and 0.02 g L^−1^ MEL to explore the effect of exogenous MEL on cold tolerance. Finally, the seedlings were pretreated with 0.01 g L^−1^ MEL before cold stress and were used for further physiological, biochemical, and molecular analysis to explore the potential mechanisms of MEL in improving cold tolerance in rapeseed.

## 2. Materials and methods

### 2.1. Plant material

In this study, a rapeseed variety (GYZ303) was used, and the seeds were supplied by the Oil Crops Research Institute, Chinese Academy of Agricultural Sciences (Wuhan, China). The seedling was cultured as described in our previous method.^9^

### 2.2. The pretreatment with exogenous melatonin

The leaves of four-leaf seedlings were exogenously sprayed with different concentrations of MEL (0.005, 0.0075, 0.01, 0.015, and 0.02 g L^−1^). There were four seedlings in each pot, and each pot was sprayed with 20 mL MEL solution as a replication. Each treatment included three pots (three replications). The control (CK) seedlings were treated with an equal amount of ddH_2_O. After the treatment, they were recovery growth under 25°C for 2 days.

### 2.3. Cold stress treatment

Three-week-old plants (four-leaf seedlings) grown at 25°C under a 16 h:8 h light:dark photoperiod on soil were treated with cold acclimation at 4°C for 1 d and were then placed in an auto programmable low temperature incubators (IN812C, Tokyo, Japan) set to 2°C and dropped 1°C h^−1^ to the desired temperature. After cold stress treatment, the plants were put at 4°C in the dark for 24 h and then transferred to 25°C for an additional 2 d. The survival rates were counted as described in our recent work.^9^ The CK and MEL-treated samples were harvested at each time point, immediately frozen in liquid nitrogen, and stored at −80°C for further analysis.

### 2.4. Measurement of physiological indexes

The above-mentioned samples were used for evaluating physiological parameters, the concentrations of soluble sugar (SS), soluble protein (SP), and proline (Pro), and the activities of superoxide dismutase (SOD), catalase (CAT), and peroxidase (POD) were measured according to the manual guidelines (Solarbio, China). The manual guidelines are freely available on the Solarbio website (http://www.solarbio.net/). The MDA content was measured using the thiobarbituric acid reaction method.^26^ All the physiological parameters were evaluated using three biological replications and a spectrophotometer microplate reader (Epoch, BioTek, USA).

### 2.5. Gene expression analysis

To analyze the gene expression, total RNA was extracted using thePlant RNA Mini Kit (Tiangen DP411, Beijing, China) according to the manufacturer’s instructions, and was subsequently synthesized into cDNA following the kit method(K1622, Thermo, USA) . The qRT-PCR reaction was carried out by ChamQTMSYBR® color qPCR Master Mix (High ROX Premixed) (Vazyme, Nanjing, China). The primers used for qRT-PCR were reported in our recent work,^8^ and *B. napus actin* gene was applied as an internal control. The qRT-PCR was performed following the reported method.^8^

### 2.6. Statistical data analysis

All the trials were carried out with a completely randomized design (CRD), and three biological replications. The datasets were analyzed by analysis of variance (ANOVA), and the mean variations were assessed by a Duncan’s multiple range test (DMRT) in SPSS Statistics 25.0 software (SPSS Inc., Chicago, IL). Pearson’s correlation analysis was completed using the mcor function, and corrplots were prepared using corrplot package Ver. 0.89 in RStudio.^27^ Principal component analysis (PCA) was completed using fviz-pca function of the factoextra R package Ver. 1.0.7 in RStudio. Differences at P < .05 were thought to be significant, and differences at P < .01 were thought to be highly significant.

## 3. Results

### 3.1. Effects of exogenous melatonin on the survival rate under cold stress

**Figure 1.**
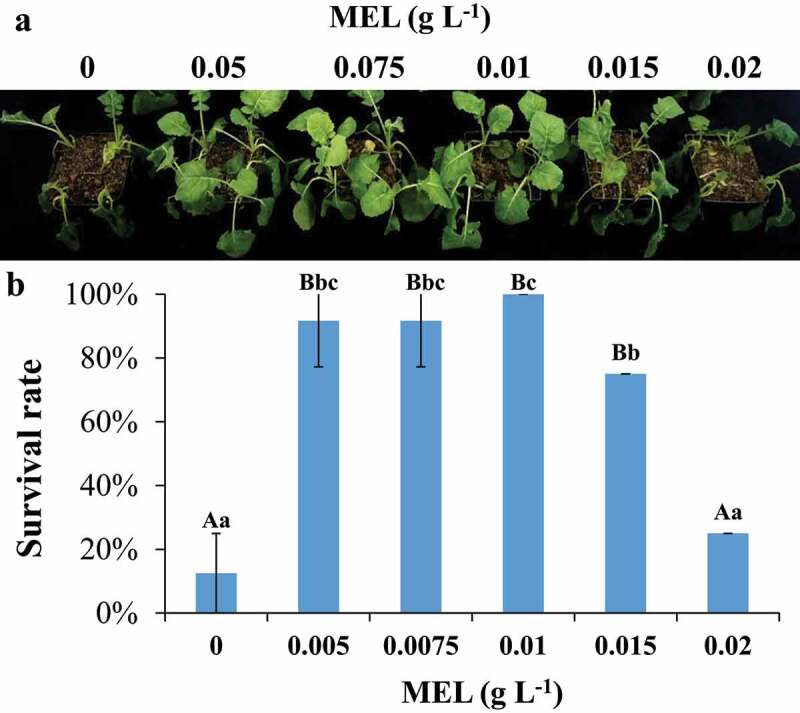
The seedlings were pretreated with melatonin under cold stress. (A) Freezing phenotypes of 3-week-old wild-type seedlings supplemented with or without melatonin. (B) The survival rates of 3-week-old wild-type seedlings supplemented with or without melatonin. The data are the means of three replicates ± SD (n = 3). Uppercase letter indicate significant differences (P < .01) and lowercase letters indicate significant differences (P < .05). Significant differences were identified using one-way ANOVA.

The seedlings were pretreated with various MEL concentrations such as 0, 0.005, 0.0075, 0.01, 0.015, and 0.02 g L^−1^, respectively, and then treated with cold stress. The results showed that the seedlings died when subjected to freezing stress (Figure 1). Under freezing stress, the survival rate of CK seedlings was 12.5%, while that pretreated with 0.005, 0.0075, 0.01, 0.015 and 0.02 g L^−1^ MEL was 91.7%, 91.7%, 100%, 75% and 25%, respectively. With the increase of MEL concentration, the survival rate of seedlings at −2°C showed an increasing and then decreasing trend in terms of survival rate (Figure 1). The survival rate of seedlings sprayed with 0.01 g L^−1^ MEL was 100% at −2°C, suggesting that the optimal dose of MEL was 0.01 g L^−1^. Thus, the rest of the analysis was performed using the samples treated with optimal dose (0.01 g L^−1^ MEL).

### 3.2. Effects of melatonin on physiological characters parameters under cold stress

**Figure 2.**
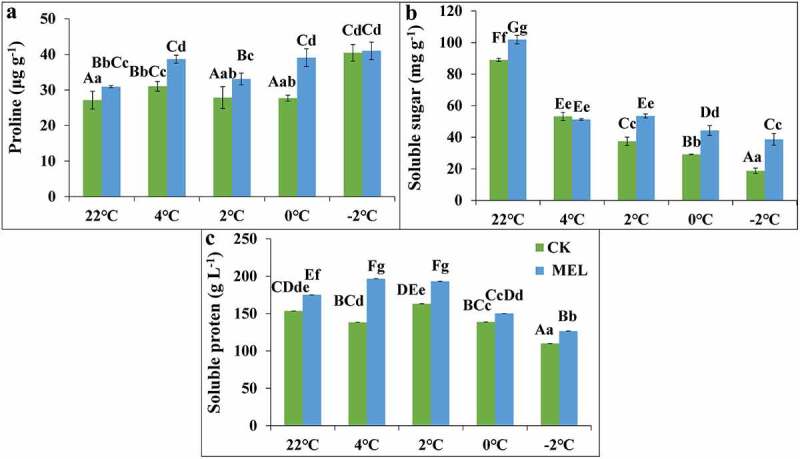
Effects of melatonin (MEL) on the content of osmoregulation substances in rapeseed under cold stress. To detect the change of osmotic regulatory substances, Guanyouza303 rapeseed seedlings pretreated with 0.01 g L^−1^ MEL were sampled at 4°C for 24 h, 2°C for 1 h, 0°C for 1 h, and −2°C for 1 h, respectively, (A) The proline content; (B) the soluble sugar content; and (C) the soluble protein content. Uppercase letters indicate significant differences (P < .01), and lowercase letters indicate significant differences (P < .05). Significant differences were identified using one-way ANOVA.

Osmoregulation substances mainly include soluble sugar, soluble protein, and proline. In this study, the seedlings were pretreated with MEL (0.01 g L^−1^), and the control group was treated with cold stress. Before and after treatment at 4°C, 2°C, 0°C and −2°C, the seedling leaves were taken to evaluate the changes in osmotic substances. When the temperature decreased from 25°C to 4°C, 2°C, 0°C, the proline content of the control increased first and then decreased. The proline content of the control was the highest at 4°C, but it increased again when the temperature decreased to −2°C. The proline content of seedlings pretreated with MEL showed a continuously increasing trend under cold treatment. Compared with the control group, the proline content of seedlings pretreated with MEL increased significantly by 14%, 26%, 19% and 41% at 22°C, 4°C, 2°C and 0°C, respectively (Figure 2A).

With the decrease of temperature, the soluble sugar content of control and MEL pretreated seedlings decreased gradually but compared with the control group, the soluble sugar content of treated rape seedlings increased significantly by 1.1, 1.4, 1.5, and 2.1 times under 22°C, 2°C, 0°C and −2°C (Figure 2B). With the decrease of temperature, the soluble protein content seedlings pretreated with MEL and water increased first and then decreased. Compared with the control group, the soluble protein content of seedlings pretreated with MEL increased by 8–42% (Figure 2C). The results showed that the contents of soluble sugar, soluble protein, and proline were improved by MEL treatment, which alleviates the damage of cold stress on rapeseed seedlings.MEL significantly improved the contents of soluble protein at 4°C, proline at 0°C and soluble sugar at −2°C compared to control.

### 3.3. Effects of melatonin on MDA and antioxidant enzymes under cold stress

**Figure 3.**
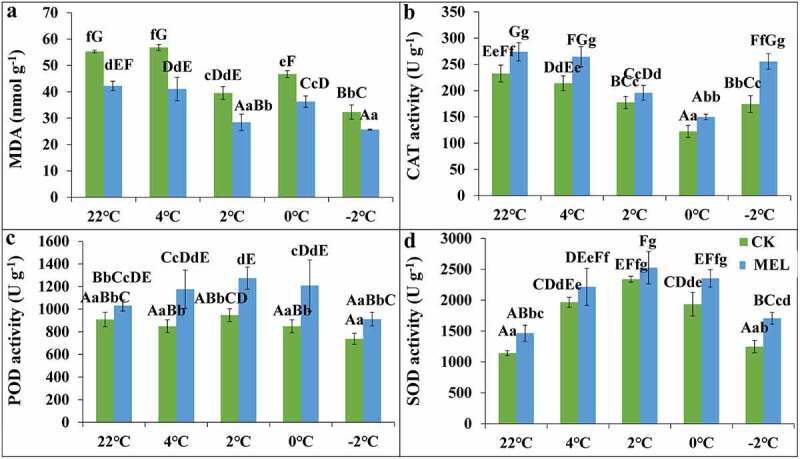
Effects of melatonin (MEL) on the MDA content and the antioxidant enzyme activities in rapeseed under cold stress. After spraying 0.01 g L^−1^ MEL, Guanyouza303 rapeseed seedlings growing at 22°C for 21 days were sampled at 4°C for 24 h, 2°C for 1 h, 0°C for 1 h, and −2°C for 1 h, respectively, to detect the changes in MDA content (A); the activity of CAT (B); the activity of POD (C); the activity of SOD (D). Uppercase letters indicate significant differences (P < .01), and lowercase letters indicate significant differences (P < .05). Significant differences were identified using one-way ANOVA.

The results showed that the MDA content of control and MEL pretreated seedlings decreased with the decrease in temperature. Compared with the control, the content of MDA in the seedlings of rape seedlings was significantly lowered than that of the control, which was 72–79% of the control (Figure 3A). MEL can alleviate the membrane lipid peroxidation of rape seedlings under cold stress.

The CAT, POD, and SOD activities were detected. When the temperature decreased from 22°C to −2°C, the CAT activity of the control group and the pretreatment of MEL decreased first and then increased. Compared with the control group, the CAT activity in the seedlings after melatonin application increased by 10–46%, and increased by 46% at −2°C (Figure 3B).

With the decrease in temperature, the POD activity of seedlings pretreated with MEL increased first and then decreased. When the temperature dropped to 4°C, 2°C, 0°C, and −2°C, the POD activity of seedlings pretreated with MEL was improved by 38%, 34%, 42% and 23% than that of control group, respectively. However, the POD activity of MEL-treated seedlings was sharply increased at 0°C (Figure 3C).

With the decrease in temperature, the SOD activity of the control group and MEL pretreated seedlings showed a trend of first increasing and then decreasing. Compared with the control group, the SOD activity of MEL pretreated seedlings increased by 8–37%, respectively. The SOD activity of seedlings pretreated with MEL was significantly higher than that of the control group, which was 1.37 times of the control group under −2°C(Figure 3D).

The POD activity of seedlings pretreated with MEL improved at 0°C, CAT and SOD at −2°C. Overall, the results suggested MEL could improve cold tolerance by increasing the activities of CAT, POD, and SOD in rapeseed.

### 3.4. Effects of melatonin on antioxidant-related genes of seedlings under cold stress

**Figure 4.**
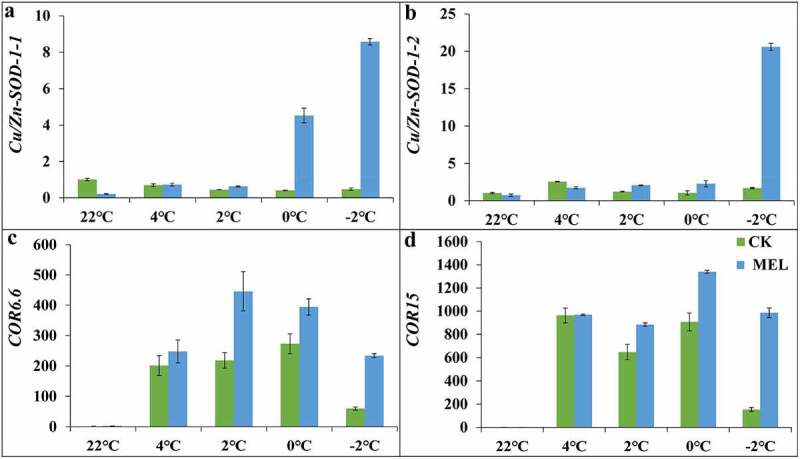
Effects of melatonin (MEL) on the gene expression under cold stress. (A) *Cu/Zn-SOD-1-1*; (B) *Cu/Zn-SOD-1-2*; (C) *COR6.6*; (D) *COR15*. The data are shown as the average ± S.D of three independent batches of seedlings for each treatment.

In this study, the *Cu/Zn-SOD* gene expression was quantitatively analyzed. The expression of *Cu/Zn-SOD-1-1* in MEL pretreated seedlings under normal conditions was only 0.2 times that of the control treatment. Under cold stress, the expression continued to rise, reaching 0.7, 0.6, 1.2, 4.5 and 8.6 times that of the control group under 4°C, 2°C, 0°C and −2°C, respectively (Figure 4A). The expression patterns of *Cu/Zn-SOD-1-2* and *Cu/Zn-SOD-1-1* in MEL pretreated seedlings under cold stress were consistent. The expression level of *Cu/Zn-SOD-1-2* in MEL pretreated seedlings increased continuously to 20.6 times compared to the normal condition, and the highest expression level of *Cu/Zn-SOD-1-2* in the control group increased to 2.5 times of that under normal conditions (Figure 4B). These results indicated that MEL maintained the balance of ROS by promoting the expression of antioxidant genes under cold stress.

### 3.5. Effects of melatonin on cold-related marker genes under cold stress

Moreover, the expression levels of cold-related marker genes such as *COR6.6* and *COR15* were also detected. The expression pattern of *COR6.6* in the control and MEL pretreatment group was increased and then decreased when the temperature dropped from 22°C to −2°C. The expression of *COR6.6* in the control group under cold stress was 247.6–445.7 times of that in the control group, while the expression of *COR15* in the MEL pretreated seedlings under cold stress was 883.6–1339.8 times of that in the control group (Figure 4C and D). . These results indicated that MEL increased cold stress by inducing the expression of *COR6.6* and *COR15* genes under cold stress.

**Figure 5.**
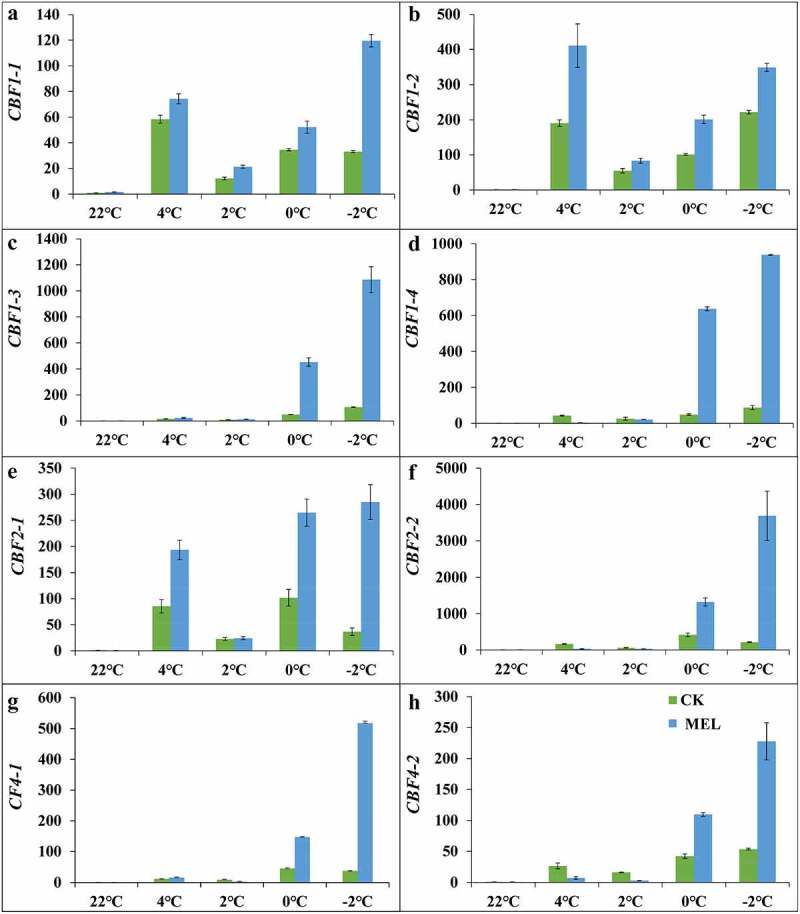
Effects of melatonin (MEL) on the *CBF* gene expression under cold stress. (A–D) Relative expression of *CBF1*; (E–F) Relative expression of *CBF2*; (G–H) Relative expression of *CBF4*. The data are shown as the average ± S.D of three independent batches of seedlings for each treatment.

In this study, the expression of all *CBF* genes was quantitatively analyzed. Under cold stress, the expression of *CBF1-1, CBF1-2*, and *CBF1-3* genes in the control group and MEL pretreated seedlings were showed an up-down-up trend, reaching 21.3–119.6, 83.3–348.7, 13.5–1086.5 times of the control group(Figure 5A–C)., After MEL pretreatment, the expression of the *CBF1-4* gene in the seedlings showed a continuously increasing trend, reaching 3.8–938.3 times of the control group (Figure 5D).

Under cold stress, the expression of *CBF2-1* gene in the MEL pretreatment group had a similar expression trend to *CBF1-1* gene, reaching 24.3–285.0 times of the control group (Figure 5E).The expression pattern of the *CBF2-2* gene in the MEL pretreatment group was continuously upregulated, reaching 30.6–3690.7 times of the control group (figure 5F). The expression of *CBF4-1* and *CBF4-2* genes in the MEL pretreated seedlings had similar expression trend to *CBF1-1* gene under cold stress. . After MEL pretreatment, the expression of *CBF4-1* increased by 16.1, 3.6, 147.8, and 517.4 times of the control group under 4°C, 2°C, 0°C, −2°C, the expression of *CBF4-2* increased by 7.4, 3.1, 109.6, and 227.8 times of the control group under 4°C, 2°C, 0°C, −2°C (Figure 5G and H). These results indicated that the expression of *CBFs* was significantly up-regulated by MEL treatment under cold stress.

### 3.6. Principal component analysis and correlation analysis

**Figure 6.**
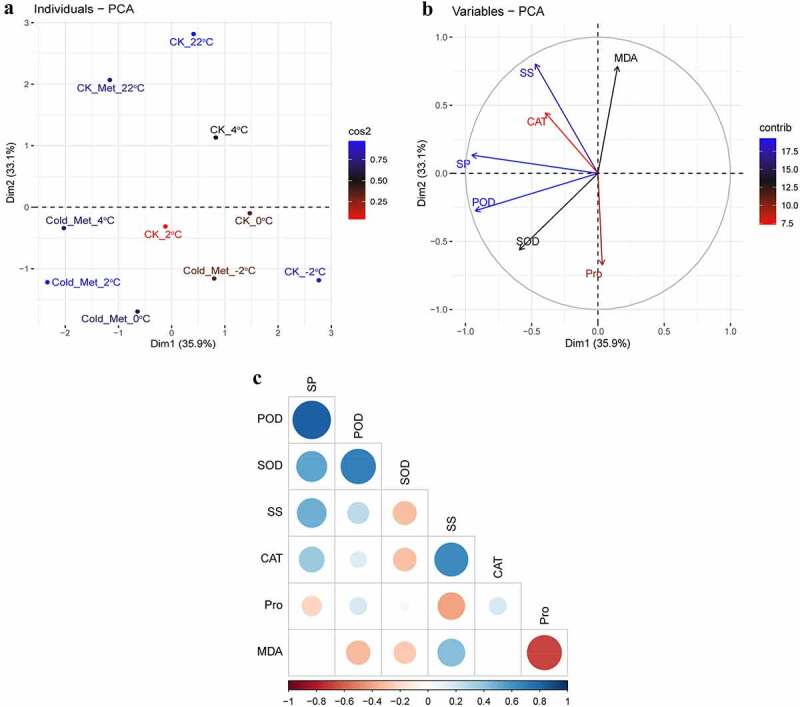
Principal component analysis (PCA) of (A) individual treatments by PCA and (B) different calculated indicators of rapeseed seedlings treated with 0.01 g L^−1^ Met. (C) Pearson’s correlation analysis between different calculated indicators of rapeseed seedlings treated with 0.01 g L^−1^ Met. The seedlings were grown at 22°C for 21 days and were sampled at 4°C for 24 h, 2°C for 1 h, 0°C for 1 h, and −2°C for 1 h. Blue to red colors indicates the strength of positive correlation. Abbreviations: superoxide dismutase (SOD), peroxidase (POD), catalase (CAT), malondialdehyde (MDA), soluble sugar (SS), soluble protein (SP), and proline (Pro).

To appraise the influence of MEL application on the studied indicators of rapeseed seedlings, the score and loading PCA diagrams were accomplished (Figure 6). The first two components, such as PC1 (35.9%) and PC2 (33.1%), showed the greatest contribution and showed 69% of the total variance in the dataset. With respect to cold stress conditions, the identical treatments at various temperatures were grouped in close proximity, while the separate treatments were split efficiently by the first two components (Figure 6A). This partition of the treatments suggested that the MEL application under cold stress had a significant enhancing influence on the studied indicators of rapeseed seedlings compared to CK. The first group of the PCA variables, e.g., PC1, was positively correlated with MDA and Pro (Figure 6B). In contrast, a noteworthy negative correlation of PC1 variables consists of SS, SP, SOD, CAT, and POD that are associated with PC2 (Figure 6B).

A Pearson’s correlation analysis was accomplished among several estimated indicators of the rapeseed seedlings (Figure 6C). The correlation analysis showed that CAT, POD, SOD, and SS were positively correlated with SP and POD; in the meantime, there were negatively correlated with MDA (Figure 6C). In short, SOD was negatively correlated with SS, CAT, and MDA; SS was negatively correlated with Pro; and MDA was negatively correlated with Pro, SOD and POD (Figure 6C). This correlation denotes a close connection between osmoprotectants and antioxidant enzymes and MEL-mediated cold tolerance in the rapeseed seedlings.

## 4. Discussion

### 4.1. Effects of melatonin on cold tolerance in rapeseed seedlings

Under cold stress, the metabolic activities of cells were limited, and the stems and leaves wilted, and became yellow and withered, resulting in growth inhibition and even plant death.^5,8,14,28^ Many studies have reported that exogenous MEL improves the cold tolerance of plants.^14,17,18^ However, the optimal concentration of MEL in improving stress tolerance varies with species. MEL (10 or 30 M) significantly increased the fresh weight, and the primary root length of *Arabidopsis thaliana* was treated at 4°C for 72 h and 120 h.^29^ Recently, many studies have reported different optimal MEL doses in different crop species and various stress conditions. In wheat, the treatment of 1 mM MEL improved cold tolerance.^30^ Cold tolerance was enhanced by 100 µM MEL in bermudagrass^31^ and that was improved by 1 mm MEL in maize.^32^ The study in melon (*Cucumis melo* L.) showed that 200 µM MEL improved cold tolerance,^33^ and pretreatment of root with 1.5 μM Mel alleviated the damage of cold treatment to watermelon.^34^ Pretreatment with 100 μmol L^−1^ MEL enhanced the adaptability of seedlings to low temperature (4°C) in *Brassica campestris* “Longyou 6”.^25^ For rapeseed seedlings, 50 μmol L^−1^ (0.01 g L^−1^) MEL significantly improved drought tolerance,^21^ and 30 μmol L^−1^ (0.007 g L^−1^) MEL significantly improved salt tolerance.^35^ The current study showed that the optimal dose of MEL in rapeseed was 50 μmol L^−1^ (0.01 g L^−1^), and the survival rate of the seedlings sprayed with MEL at −2°C increased to 100%.

### 4.2. Physiological and biochemical mechanism of melatonin-mediated improved cold tolerance of rapeseed seedlings

When plants are subjected to stress, a large number of osmotic adjustment substances are accumulated to maintain cell osmotic potential.^15,28^ For instance, Li, Zeng^21^ found that the soluble sugar, soluble protein, and proline contents were increased by MEL in rapeseed seedlings under drought conditions.^21^ Melatonin significantly increased the content of soluble protein, sugar, and proline and the SOD and POD activity of wheat seedlings at low temperatures. The plants pretreated with MEL showed lower MDA content and stronger SOD and POD activities at low-temperature.^31^ MEL improved the relative water content and the activities of SOD and CAT in maize plants at low-temperature.^32^ Melon plants pretreated MEL had more soluble protein and proline content, lower MDA content, and stronger antioxidant enzyme activity under low-temperature treatment.^33^ MEL alleviated the oxidative stress of watermelon caused by cold treatment.^34^ The plants pretreated with MEL showed more sucrose and proline content, lower MDA content, and stronger antioxidant enzyme activity.^36^ Under low-temperature treatment, rice seedlings pretreated with MEL showed lower MDA content and stronger antioxidant enzyme activity.^37^ Exogenous serotonin increased the content of soluble sugar, soluble protein and proline by 37–65%, 1–16%, and 7–38%, compared to that of the control, respectively.^8^ MEL increased the content of soluble sugar, soluble protein and proline by 14–106%, 8–42%, and 14–41% compared to that of the control, respectively. Compared with the control, the MDA content in the seedlings pretreated with serotonin was decreased by 13–30%,^8^ while decreased by 21–28% in MEL-treated seedlings. The pretreatment of serotonin increased POD, CAT, and SOD activities by 10–31%, 4–42%, 9–27%,^8^ respectively. WhereasMEL increased POD, CAT, and SOD activities by 14–42%, 10–46%, 8–37%, respectively. Compared to serotonin, MEL had more ability to improve cold tolerance in rapeseed seedlings. MEL significantly improved the content of soluble protein at 4°C, content of proline and POD activity at 0°C, content of soluble sugar, CAT and SOD activity at −2°C compared to control at −2°C. The results showed that with the decrease of temperature, MEL increased the content of soluble sugar, soluble protein, proline, and other osmotic adjustment substances, reduced the content of MDA, and improved the activities of the antioxidant defense systems to alleviate the damage of chilling stress in rapeseed seedlings.

### 4.3. Molecular mechanism of melatonin improving cold tolerance

It is found that MEL and its metabolites directly regulate the transcription level of antioxidant enzyme genes to increase stress tolerance in plants.^5,6,12^ The proportion of Cu/Zn SOD enzyme in SOD enzyme is the largest, especially in the active oxygen scavenging enzyme system, which is closely connected with cold, saline-alkali, and other stress tolerance in plants.^38,39^ Studies have shown that *ICE-CBF-COR* transcriptional network is critical in the process of cold acclimation.^40^ Melatonin regulated the gene expression of *AtCBF1, AtCBF2, AtCBF3*, and *COR15a* to enhance salt, drought and cold tolerance in Arabidopsis.^29,41^ In watermelon, the expression of *CBF* and *COR* genes was significantly up-regulated by MEL at low-temperature.^34^ Under cold treatment, MEL pretreatment promoted the expression of *CBF* in tomato.^36^ Exogenous serotonin increased the expression of Cu/Zn-SOD-1-1 by 2.2 folds in the seedlings under −2°C,^8^ MEL increased by 8.6 folds. Exogenous serotonin increased the expression levels of COR6.6 by 218.2, 426.3, 312.9 and 231.2 folds compared to that in control under 4, 2, 0, and −2°C.^8^ MEL increased by 247.6, 445.7, 394.2 and 233.7 folds compared to that in control at 4, 2, 0, and −2°C. After the cold treatment, exogenous serotonin increased the expression of CBF1-4 by 317.1 folds under −2°C,^8^ while MEL increases the expression by 938.3 folds compared to that in control under −2°C. Under cold stress, exogenous serotonin increased the expression of the *CBF2-2* gene by 14.2–2698.1 folds under cold stress,^8^ MEL increases by 30.6–3690.7 folds compared to that in control. Under cold stress, exogenous serotonin increased the expression of *CBF4-1* by 188.5 folds under −2°C,^8^ MEL increased by 517.4 folds compared to that in control under −2°C. These results showed MEL had more ability of transcriptional regulation in *Cu/Zn-SOD-1-1, COR6.6, CBF1-4, CBF2-2* and *CBF4-1* than serotonin. In our study, the gene expression analysis indicated that MEL significantly improved the gene expression of *Cu/Zn SOD, CBF*s, and *COR*s, thus improving the cold tolerance of rapeseed.

## 5. Conclusion

**Figure 7.**
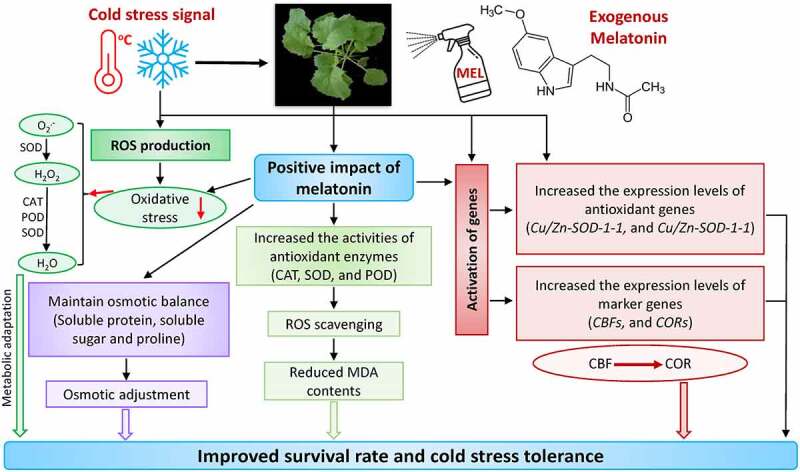
A mechanistic chart shows the mechanism of melatonin-mediated cold stress tolerance in rapeseed seedlings. Exogenous melatonin not only adjusts the antioxidant systems and osmotic regulation; however, it also substantially contributes to the activation of stress-responsive genes by regulating the signaling network to improve cold tolerance in rapeseed.

The current study concluded that exogenous MEL improved cold tolerance by sustaining the osmotic equilibrium in plant cells under cold stress by enhancing the contents of osmoprotectant substances, i.e., soluble sugar, soluble protein, and proline. Moreover, exogenous MEL also improves the ROS scavenging system by improving the activities of antioxidant defense enzymes such as CAT, POD, and SOD; and the expression level of stress-related genes (antioxidant and marker genes) in rapeseed (Figure 7). Notably, MEL also contributed to the cold stress-related signaling system by regulating the expression of *CBFs* and *CORs* (Figure 7). In the near future, more studies should be carried out to reveal the mechanisms of MEL-induced regulation of marker, *CBFs*, and *CORs* genes, which helps in improving the cold tolerance in rapeseed seedlings.
